# Costs of Illness in the 1993 Waterborne *Cryptosporidium* Outbreak, Milwaukee, Wisconsin

**DOI:** 10.3201/eid0904.020417

**Published:** 2003-04

**Authors:** Phaedra S. Corso, Michael H. Kramer, Kathleen A. Blair, David G. Addiss, Jeffrey P. Davis, Anne C. Haddix

**Affiliations:** * Centers for Disease Control and Prevention, Atlanta, Georgia, USA; † City of Milwaukee Health Department, Milwaukee, Wisconsin, USA; ‡ Wisconsin Division of Public Health, Madison, Wisconsin, USA; § Emory University, Atlanta, Georgia, USA

**Keywords:** cost of illness, costs and cost analysis, healthcare costs, hospital costs, disease outbreaks, *Cryptosporidium parvum*, cryptosporidiosis, drinking water, research

## Abstract

To assess the total medical costs and productivity losses associated with the 1993 waterborne outbreak of cryptosporidiosis in Milwaukee, Wisconsin, including the average cost per person with mild, moderate, and severe illness, we conducted a retrospective cost-of-illness analysis using data from 11 hospitals in the greater Milwaukee area and epidemiologic data collected during the outbreak. The total cost of outbreak-associated illness was $96.2 million: $31.7 million in medical costs and $64.6 million in productivity losses. The average total costs for persons with mild, moderate, and severe illness were $116, $475, and $7,808, respectively. The potentially high cost of waterborne disease outbreaks should be considered in economic decisions regarding the safety of public drinking water supplies.

*Cryptosporidium parvum*, a protozoan parasite that causes gastrointestinal illness, is transmitted by ingestion of oocysts excreted in human or animal feces. Typical modes of transmission include person to person, animal to person, by exposure to contaminated surfaces, and by ingestion of impure food or water (*1*). From 1990 to 2000, at least 10 cryptosporidiosis outbreaks associated with contaminated drinking water were reported in the United States (*2*–*5*). Although the health impact of an outbreak of cryptosporidiosis originating from a contaminated public water source has been carefully documented (*6*), little effort has been made to estimate the economic impact of such an outbreak. This study estimates the cost of illness associated with perhaps the largest outbreak associated with a contaminated public water source ever reported in the United States. In 1993, an estimated 403,000 residents of the greater Milwaukee, Wisconsin, area (population, approximately 1.61 million) became ill when an ineffective filtration process led to the inadequate removal of *Cryptosporidium* oocysts in one of two municipal water treatment plants (*6*). We assessed direct medical costs and productivity losses from diarrheal illness during the Milwaukee outbreak to estimate the total cost of illness and the average cost per person with mild, moderate, and severe illness.

This cost-of-illness analysis was based on epidemiologic data collected during and after the 1993 cryptosporidiosis outbreak in Milwaukee, Wisconsin. Primary data on utilization and cost of inpatient admissions were obtained from a review of medical and financial records from hospitals in the greater Milwaukee area.

## Methods

### Epidemiologic Burden of Illness

A telephone survey of 613 households provided estimates on the total number of persons in Milwaukee experiencing mild, moderate, or severe illness as a result of the cryptosporidiosis outbreak (*6*). Cases were defined as residents of Milwaukee County or the surrounding four counties (Washington, Ozaukee, Racine, and Waukesha) with onset of watery diarrhea from March 1 to April 28, 1993 (the outbreak period). When disease case estimates were adjusted for normal background diarrheal disease rates, investigators estimated that 403,000 residents of the five-county area experienced illness caused by the cryptosporidiosis outbreak (*6*). Of this group, an estimated 354,600 persons (~88%) did not seek medical attention; 44,000 persons (~11%) were seen as outpatients; and 4,400 persons (~1%) were hospitalized.

### Cost of Illness

Following the design of the epidemiologic studies of the same outbreak, we categorized illness as mild, moderate, or severe by type of medical care sought during the outbreak period and the following 2 months (4-month study period) when persons were still likely to seek medical care (*6*–*8*). Persons with mild illness did not seek physician or emergency department care for their illness. Persons with moderate illness had at least one physician or emergency department visit but were not hospitalized. Persons with severe illness were hospitalized at least once during this period.

Previous studies and evidence collected during the outbreak suggest that underlying medical conditions such as AIDS can increase the severity of illness in persons infected with *Cryptosporidium* (*9*,*10*). To capture the effect of underlying condition on cost of illness, we further classified patients with moderate and severe illness as having no underlying condition, an underlying condition likely treated with immunosuppressive drugs, or AIDS.

Data on utilization and average cost of inpatient services, emergency department visits, ambulance transports, and medication for persons with moderate and severe illness were obtained from a review of medical and financial records from 11 of the 14 hospitals in the greater Milwaukee area. The three nonparticipating hospitals did not differ in the number of confirmed cases of persons infected with *Cryptosporidium,* nor did they serve specialty populations that would result in higher medical costs per case. Total cost of illness was estimated from average cost of illness multiplied by the burden of illness. All clinical and financial data were recorded on standardized forms and entered into a computerized database. We did not collect information that identified patients by name or billing account number. Additional data on use of services and costs for persons with mild illness and data on productivity losses were obtained from the City of Milwaukee Health Department and published epidemiologic studies on the outbreak (*6*–*8*).

Cost-of-illness estimates for mild, moderate, and severe illness included both direct medical costs and indirect costs associated with lost productivity. Medical costs included costs for inpatient and outpatient health services, ambulance transport, and medication. Productivity losses included time lost by infected persons due to illness and the time lost by caregivers or family members to tend to ill persons. All costs are presented in 1993 U.S. dollars. We did not include litigation costs, the cost of preventive measures (e.g., switching to bottled water), intangible costs associated with pain and suffering, or the cost to the local, state, and federal government to investigate and control the outbreak.

### Medical Costs

We used several parameters to estimate the direct medical costs associated with diarrheal illness during the Milwaukee cryptosporidiosis outbreak (Table 1).

**Table 1:** Parameter estimates for calculating the cost of diarrheal illness during the cryptosporidiosis outbreak, Milwaukee, Wisconsin, 1993

Parameter	Mild illness^a^	Moderate illness	Severe illness
% of infected persons with diarrhea	87.99 (6)	10.92 (6)	1.09 (6)
**Medical costs**			
Inpatient healthcare costs			
% with hospitalization			100
Cost of hospitalization	–	–	$6,312^b^
Outpatient healthcare costs			
% with physician visit		95^c^	29^b^
Cost of physician visit	–	$45^d^	$45^d^
% with emergency department visit		5^b^	
Cost of emergency department visit	–	$224^b^	
Ambulance transport			
% of emergency department visit/hospitalization with ambulance transport	–	4.9^b^	16.3^b^
Cost of ambulance transport	–	$228 (11)^c^	$228 (11)^c^
Medication			
Self-medication before seeking healthcare			
Duration of illness before seeking health care, mean days	4.7^e^	5.8^b^	18.4^b^
% taking medication	30^c^	30^b^	29^b^
Cost of medication	$5.73 (12)^c^	$5.92 (12)^c^	$6.74 (12)^b^
Medication prescribed after seeking healthcare			
% taking medication	—	54^b^	48^b^
Cost of medication		$8.91 (12)^b^	$70.52 (12)^b^
Recurrent episodes			
% with recurrent episodes (and assuming 100% take medication)	21 (7)	21 (7)	21 (7)
Duration of recurrent episodes, mean d	2 (8)	2 (8)	2 (8)
Cost of medication	$2.44 (12)^c^	$2.44 (12)^c^	$2.44 (12)^c^
**Productivity losses**			
Productivity losses for ill persons, mean d	1.3^e^	3.8^e^	13.5^e^
Productivity losses for caregivers, mean d	0.1^e^	1.3^e^	3.9^c^
Earnings lost/d	$81 (13)	$81 (13)	$81 (13)

^a^Reference nos. are in parentheses. ^b^Medical chart review. ^c^Author assumption. ^d^City of Milwaukee Health Department. ^e^Random digit dial survey conducted by the City of Milwaukee Health Department.

### Inpatient and Emergency Department Health Care Costs

To assess the usage and average cost of inpatient admissions (e.g., hospitalizations) and outpatient services associated with emergency department visits, we reviewed all hospital medical charts for persons with laboratory-confirmed cryptosporidiosis as identified by the hospitals’ laboratory records. Because the sensitivity of diagnostic testing is relatively low, and many persons were not tested during the outbreak, we also reviewed a sample of charts for persons admitted to the emergency department or hospital with diarrhea for at least 2 days, as identified by the following diagnostic codes from the International Classification of Diseases, 9th Revision, Clinical Modification (CD-9-CM) listed in one of the first four diagnosis categories on the hospital discharge record: 007.20, 008.80, 009 and subcategories, 079.90, 234.10, 276 and subcategories, 558.90, and 999 and subcategories. During these admissions, either no laboratory testing was performed or tests were negative for *Cryptosporidium* and other intestinal pathogens. For the two samples, we included all costs for the inpatient admission or emergency department visit, regardless of whether the cost was directly attributable to cryptosporidiosis. Charts were excluded when the hospital admission was primarily for another condition (i.e., ICD-9-CM codes were not listed in one of the first four diagnostic categories) and when the onset of gastrointestinal illness occurred after hospital admission.

From the medical records, we collected data on resource use during hospitalization or emergency department visit, the use of ambulance transportation, self-reported use of medication before an emergency department visit or inpatient admission, and physician-prescribed medication following an emergency department visit or inpatient admission. Charges for hospitalization included diagnostic, laboratory, hospital room, and technical services (e.g., physical therapy, occupational therapy, and respiratory services); attending physician and nursing staff; medication; emergency department services; and other supplies or services not identified in the previous categories (e.g., medical-surgical supplies, clinic services).

Charges for emergency department visits and hospitalizations were converted to costs by using an average cost-to-charge ratio of 0.67, based on ratios obtained from 6 of the 11 hospitals sampled in the greater Milwaukee area; this figure is comparable to Wisconsin’s average operating cost-to-charge ratio (0.70) reported for urban hospitals in 1993 (*14*). Charges for specialty consultations not included in the hospital bill were excluded from this analysis because insufficient data were available on the number, duration, or charges for these services.

### Outpatient and Ambulance Costs

We assumed that 95% of persons with moderate illness sought the care of a physician (one visit only) and that the remaining 5% required an emergency department visit. (Data collected from the epidemiologic investigation provided information on whether ill persons sought healthcare for their illness and whether they were hospitalized. No information was collected on whether a nonhospitalized healthcare visit was to seek physician or emergency department services. Therefore, in the absence of reliable data, we assumed that 5% of persons with moderate cryptosporidiosis went to the emergency department). For the latter group, we assumed that no additional physician visits were needed before or after the emergency department visit. The proportion of persons with severe illness who had a physician visit before hospitalization was obtained from chart review. We assumed that one physician visit was needed before (and none after) the hospitalization. The cost of a physician visit ($45) was obtained from data collected by the City of Milwaukee Health Department. (Since costs, and not charges, for physician visits were provided, obtaining cost-to-charge ratios was not necessary.) This figure is in the range found by other studies that have estimated the cost of a physician visit as ranging from $40 (1992 dollars) to $53 (1994 U.S. dollars) during this period (*15*,*16*).

Use of ambulance transport was indicated on the medical charts for emergency department visits and hospitalizations. Ambulance transport was used by 4.9% of those with moderate illness involving an emergency department visit compared with 16.3% of those with serious illness. We used the 1993 rate set by the City of Milwaukee ($185.50 for conveyance, $12 for minor services, and $6 per mile) for the cost of an ambulance transport, and we assumed that the average distance per transport was 5 miles (*11*).

### Medication Costs

For mild illness, data regarding the duration of illness were collected by the City of Milwaukee Health Department by using a random digit dial survey. Methods for this data collection were published (*6*). For moderate and severe illness, data regarding the duration of illness before an emergency department visit or hospitalization, the percentage of persons self-medicating during this period, and costs for medication were obtained from the medical records. We assumed the percentage of persons who self-medicated, as obtained from emergency department records for a person with moderate illness, also applied to persons with mild illness and to persons who did not use the emergency department but sought other medical care.

We estimated that all persons with mild illness who self-medicated used four 2-mg tablets of loperamide antidiarrheal medication per day or two 32-oz packs of oral rehydration solution per week, at a cost of $2.44/d. In the absence of reliable data on the duration of self-medication for a person with mild illness, we assumed that persons took medication for 50% of the duration of illness.

From the medical records, we collected detailed drug information (i.e., type, quantity, and duration) for medications prescribed upon discharge for persons with moderate and severe illness. We assumed that medications prescribed for persons with moderate illness seeking an emergency department visit also applied to persons with moderate illness seeking physician care. Retail drug prices in 1993 were used to calculate all costs (*12*).

Data about recurrent illness for mild, moderate, and severe illness were obtained from two investigations conducted during the outbreak (*7*,*8*). On the basis of these data, we estimated that 21% of ill persons experienced a recurrent episode of diarrhea for 2 days. As we did for persons with mild illness, we assumed that persons with recurrent illness took medication for 50% of the duration of illness at a cost of $2.44/d.

### Productivity Losses

Productivity losses for ill persons and their caregivers were estimated from data on days lost because of illness collected by the random digit dial survey conducted by the City of Milwaukee Health Department (*6*). In the absence of reliable data on the days lost by caregivers of persons with severe illness, we assumed that a caregiver was needed for 50% of the number of days hospitalized. The value of missed work time by a caregiver or person with diarrheal illness was estimated by using the average annual wages for residents of Wisconsin in 1993 (*13*), increased by 25% to include fringe benefits. Because the type of day lost (i.e., work or leisure) was not specified in the secondary data available, we used an average daily value of $81 (annual wage plus fringe benefits, divided by 365 days) (*17*). We valued the time of all persons based on the productivity of the average worker, regardless of the work force status of any person.

## Results

We reviewed approximately 2,000 medical records from October 30 through November 11, 1995, and identified 378 persons who met our case definition for a moderate or severe case of cryptosporidiosis. We collected data on 155 persons who met our case definition for a moderate illness (i.e., emergency department visit only) and 223 persons who met our case definition for severe illness (i.e., a hospitalization). Seventeen percent of persons with moderate illness and 63% of persons with severe illness in our sample had laboratory-confirmed cryptosporidiosis.

Average costs of illness for persons with mild, moderate, and severe illness were $116 for mild, $475 for moderate, and $7,808 for severe (Table 2). Direct medical costs represented 2% of the average cost for persons with mild illness, 13% of the average cost for persons with moderate illness, and 82% of the average cost for persons with severe illness. The average cost of illness for all persons who experienced diarrheal illness, weighted by the proportion in each illness category, was $239 per person: $79 in medical costs and $160 in productivity losses.

**Table 2:** Average cost per person with mild, moderate, and severe illness^a,b^

Illness severity	Medical costs ($)	Productivity losses ($)	Total ($)
Mild	2	113	116
Moderate	62	413	475
Severe	6,399	1,409	7,808
Average cost of illness	79	160	239

^a^Costs in 1993 United States dollars. ^b^Costs may not add up due to rounding

The total cost of illness associated with the cryptosporidiosis outbreak in Milwaukee was approximately $96.2 million: $31.7 million in direct medical costs and $64.6 million in productivity losses (Table 3). Medical costs accounted for 33% of the total cost of illness, including $790,760 for mild illness, $2.7 million for moderate illness, and $28.2 million for severe illness. Productivity losses accounted for 67% of the total cost of illness, including $40.2 million for mild illness, $18.2 million for moderate illness, and $6.2 million for severe illness. Nearly 43% of all costs were attributable to persons with mild illness, 22% to persons with moderate illness, and 36% to persons with severe illness.

**Table 3:** Total cost of illness during the 1993 cryptosporidiosis outbreak^a,b^

Illness severity	Medical costs ($)	Productivity losses ($)	Total ($)
Mild (n=354,600)	790,760	40,212,000	41,002,000
Moderate (n=44,000)	2,710,800	18,176,000	20,887,000
Severe (n=4,400)	28,153,000	6,201,400	34,355,000
Total cost of illness (n=403,000)	31,655,000	64,589,000	96,244,000

^a^Costs in 1993 United States dollars. ^b^Costs may not add up due to rounding.

### Costs for Emergency Department Visits and Hospitalizations by Underlying Condition

The average cost for an emergency department visit for persons with moderate illness was $224 (Table 4). For persons with no underlying condition (84% of emergency department visits), the average cost for an emergency department visit was $213. For persons with underlying conditions (only one patient had AIDS), the average cost for an emergency department visit was $265. The average cost for a hospitalization for persons with severe illness was $6,312, with an average length of stay of 8 days. For persons with no underlying condition (34% of hospitalizations sampled), the average cost for a hospitalization was $3,131, with an average length of stay of 5 days. For persons with an underlying condition other than AIDS (52% of hospitalizations that met the case definition), the average cost for a hospitalization was $5,520, with an average length of stay of 7 days. Persons with AIDS (14% of hospitalizations) incurred the greatest average cost of hospitalization, $17,388, with an average length of stay of 16 days.

**Table 4:** Average cost for emergency department visits and hospitalizations, by underlying condition^a^

	Emergency department visit ($)	Hospitalization ($)	Average length of stay (d)
No underlying condition	213	3,131	5
Underlying condition, other than AIDS	265	5,520	7
AIDS	NA	17,388	16
All conditions	224	6,312	8

^a^Costs in 1993 United States dollars. ^1^In 1998, the City of Milwaukee completed an $89 million renovation of two municipal water treatment plants, which together serve approximately 800,000 people, as part of an effort to control future outbreaks of C*ryptosporidium* (*22*).

## Discussion

The massive waterborne outbreak of cryptosporidiosis in 1993 in Milwaukee caused illness in approximately 403,000 persons and generated substantial healthcare costs and productivity losses. We estimate that on average, ill persons incurred approximately $79 in medical costs and $160 in productivity losses, resulting in total medical costs of $31.7 million in total medical costs and $64.6 million in total lost productivity. Since epidemiologic estimates of incidence contribute substantially to total cost estimates for any outbreak, information on average cost of illness by severity can be applied to any range of epidemiologic estimates to assess the sensitivity of total costs. For example, in the Milwaukee outbreak, the 95% confidence interval for burden (incidence or prevalence) of illness ranged from 370,000 to 435,000 persons (2,400 to 6,400 for severe cases, and 38,000 to 50,000 for moderate cases) (*6*). Applying these epidemiologic burden of illness estimates to the average cost per case by severity, total medical costs and productivity losses for the Milwaukee outbreak ranged from $75 to $118 million.

Although only 1% of persons who experienced diarrheal illness associated with the outbreak were hospitalized, their medical costs accounted for 89% of the total outbreak-related medical costs. Persons with suppressed immune systems were the most severely affected, accounting for 66% of hospitalizations and 74% of the total outbreak-related direct medical costs. Persons with AIDS incurred hospital costs five times greater than persons with no underlying condition. Persons with underlying conditions other than AIDS incurred almost twice the cost of hospitalization compared with persons with no underlying condition.

During the 4-month period during and after the outbreak, the productivity of Milwaukee residents and visitors who experienced diarrheal illness and their caregivers was severely affected. Although mild illness did not represent a great strain on the use of medical care resources, productivity losses were substantial given the number of persons who experienced mild illness that debilitated them in some capacity. Productivity losses accounted for 98% of total costs for persons with mild illness and 87% of total costs for persons with moderate illness.

The cost-of-illness estimates in this study are conservative for several reasons. Primary data collection from medical and financial records limited our ability to assess all costs associated with the outbreak. For example, medical and financial records lacked details about physician visits, ambulance transports, or self-medication before admission, and cost information for professional services provided during hospitalization that were billed separately. Further, an estimate of the magnitude of the occurrence of illness among visitors to the greater Milwaukee area was not made. Conservative estimates were used for any assumptions made when reliable data were not available. Second, we excluded productivity losses associated with chronic illness that might have extended beyond our 4-month study period, and we also excluded productivity losses associated with premature death. An estimated 69 deaths occurring principally among persons with AIDS were attributed to Milwaukee’s cryptosporidiosis outbreak (*18*). Excluding the productivity losses associated with premature mortality potentially underestimates our results for total productivity losses associated with the outbreak.

While this study focused on the direct medical costs and productivity losses for illness associated with the outbreak, a broader perspective for the analysis would have included other nonmedical costs for infected persons, costs to businesses, and the cost to government agencies of controlling the outbreak and improving the public water system. Costs to government agencies alone, including the Centers for Disease Control and Prevention (CDC), the Environmental Protection Agency, the Wisconsin Division of Health (currently the Wisconsin Division of Public Health), the City of Milwaukee Health Department, the Milwaukee Water Works, and 17 local health departments, were estimated at >$2 million immediately following the outbreak (CDC, unpublished data). A class action suit filed by the residents of Milwaukee against the city continued to generate costs for the local government well beyond the immediate outbreak period. Businesses similarly experienced financial hardship during the outbreak because of employee illness, the necessity of using bottled water during the city’s boil-water advisory, and a decrease in beverage and food sales overall. Unaccounted costs for the infected person include costs incurred for self-protection (i.e., the purchase of bottled water) and the pain and suffering associated with illness.

Although the $96.2 million in illness costs attributed to the Milwaukee outbreak is substantial, estimated monetized annual costs of waterborne disease in the United States have been estimated at $21.9 billion (1991 dollars) (*19*). This figure is based on estimates of 7.1 million cases of mild to moderate waterborne disease and 560,000 cases of severe disease (*20*), and an average cost per case of $2,860, including medical costs and productivity losses (*21*). (Average cost per case, $2,860, is based on a study of a giardiasis outbreak in Pennsylvania in 1983–1984 [*21*]. Although the case-fatality ratio was lower than in Milwaukee, the cost per case was higher than our estimates because of a longer duration of illness. The authors [*19*] note that $2,860 likely overestimates the cost of a mild case and underestimates the cost of a severe case.)

In an era of limited health resources, decision makers must choose how to allocate resources to improve the public’s health. Measures taken to reduce the risk of waterborne cryptosporidiosis will also prevent other waterborne diseases. Water authorities often face the predicament of dealing with decreasing raw water quality, the high costs of new technologies, water filtration systems that do not completely remove all potentially pathogenic organisms, and increased public demand for safe water. The cost of this outbreak, which can be balanced against the cost of measures for preventing future outbreaks,^1^ is a reminder that failure to maintain safe drinking water supplies has substantial impact on the health and economy of a community.
